# Nasal Versus Conjunctival Allergen Provocation Tests: Diagnostic Performance and Clinical Utility in Respiratory Allergy

**DOI:** 10.7759/cureus.111597

**Published:** 2026-06-27

**Authors:** Maria Tsami, Christelos Kapatais, Nikolaos Syrigos, Andriana I Papaioannou, Petros Bakakos, Ekaterini Syrigou, Nikoleta Rovina

**Affiliations:** 1 Allergy Unit, Sotiria Chest Diseases Hospital, Athens, GRC; 2 1st Department of Respiratory Medicine, National and Kapodistrian University of Athens Medical School, Sotiria Chest Diseases Hospital, Athens, GRC; 3 3rd Department of Internal Medicine and Laboratory, National and Kapodistrian University of Athens Medical School,Sotiria Chest Diseases Hospital, Athens, GRC; 4 3rd Department of Internal Medicine and Laboratory, National and Kapodistrian University of Athens Medical School, Sotiria Chest Diseases Hospital, Athens, GRC

**Keywords:** allergen provocation tests, component-resolved diagnostics, conjunctival allergen provocation testing, decision curve analysis, diagnostic accuracy, nasal allergen provocation testing, polysensitization, rhinitis

## Abstract

Background: Respiratory allergic diseases are highly prevalent and often underdiagnosed, requiring diagnostic approaches that reflect clinically relevant allergic responses. Conventional methods, including skin prick tests (SPTs), serum-specific immunoglobulin E (sIgE), and component-resolved diagnostics (CRD), primarily assess sensitization and may not consistently correlate with clinical reactivity.

Methods: Eighty participants (50 patients with respiratory allergy and 30 controls) underwent SPTs, sIgE, CRD, nasal allergen provocation testing (NAPT), and conjunctival allergen provocation testing (CAPT). Diagnostic indices were calculated. Multivariable logistic regression identified predictors of positive provocation tests. Model performance was assessed using receiver operating characteristic (ROC) curves with bootstrap-derived confidence intervals, calibration analysis (locally weighted scatterplot smoothing), and decision curve analysis (DCA).

Results: CAPT demonstrated higher sensitivity than NAPT across all reference standards (91.3-94.9% vs 75.0-86.1%). In contrast, NAPT showed superior discriminative performance, diagnostic accuracy, and model calibration. Polysensitization and asthma were the strongest predictors of positive NAPT responses. ROC analysis showed good discrimination, particularly for *Olea europaea* (AUC 0.875). DCA indicated greater net clinical benefit for the NAPT-based model across relevant threshold probabilities. Agreement analysis suggested that provocation tests capture aspects of clinical reactivity not fully reflected by sensitization-based diagnostics.

Conclusions: CAPT demonstrates high sensitivity, whereas NAPT provides greater clinical discrimination and decision-making value, particularly in complex allergic phenotypes. NAPT may offer clinically actionable information in patients with discordant or polysensitized profiles, supporting allergen selection and optimization of immunotherapy strategies.

## Introduction

Respiratory allergic diseases represent one of the most widespread chronic conditions worldwide [1-4] and are associated with impaired daily functioning, reduced work performance, and substantial socioeconomic burden [5-9]. A considerable proportion of affected individuals remain undiagnosed with allergic rhinitis (AR) or allergic conjunctivitis (AC) despite the presence of suggestive clinical symptoms, highlighting the need for accurate and clinically meaningful diagnostic approaches.

The evaluation of suspected respiratory allergy typically combines in vivo testing, including skin prick tests (SPTs), in vitro assessment of serum-specific immunoglobulin E (sIgE), and molecular component-resolved diagnostics (CRD), which improve sensitization profiling [10-14]. However, these methods primarily reflect immunological sensitization and may not always correspond to clinically relevant allergic disease, particularly in polysensitized patients or in cases with discordance between test results and clinical history [15,16].

Additional diagnostic methods include specific nasal allergen provocation testing (NAPT) and conjunctival allergen provocation testing (CAPT), which help identify the allergens responsible for clinical reactivity, especially in complex cases [17,18]. By directly assessing the response of target organs, these tests provide functional information that complements conventional diagnostic modalities.

Both NAPT and CAPT have been demonstrated as useful tools in the diagnosis of different types of rhinitis, including seasonal and perennial AR [17,18], local AR and conjunctivitis [19-21], and occupational rhinitis [22,23]. Furthermore, they contribute to a better understanding of the underlying pathophysiological mechanisms [24-27] and support allergen selection and evaluation of immunotherapy effectiveness [28-31].

Despite the availability of multiple diagnostic modalities, the optimal integration of sensitization-based and functional testing in routine clinical practice remains uncertain. In particular, the diagnostic performance and clinical utility of CAPT relative to NAPT, SPT, sIgE, and CRD remain incompletely characterized, especially in polysensitized patients and real-world clinical settings.

The present study aimed to provide a comprehensive evaluation of nasal and conjunctival allergen provocation tests in patients with respiratory allergy classified using conventional and molecular diagnostic methods. The primary objective was to assess the diagnostic performance of NAPT and CAPT relative to established diagnostic modalities, including SPTs, sIgE, and CRD. Secondary objectives were to evaluate agreement between diagnostic methods, assess predictive values, examine model discrimination and calibration characteristics, and explore the potential complementary role of provocation testing in clinically relevant subgroups, including polysensitized patients. Advanced statistical approaches, including multivariable modeling and decision curve analysis (DCA), were applied as exploratory analyses to further investigate factors associated with positive provocation test responses and to assess model performance within the study population. By integrating functional testing with real-world sensitization profiles, this study sought to clarify the complementary roles of NAPT and CAPT in the diagnostic evaluation of respiratory allergy.

## Materials and methods

This was a prospective observational diagnostic accuracy study that was conducted at the Allergy Department and the 1st Department of Respiratory Medicine, in Sotiria Chest Hospital, National and Kapodistrian University of Athens, Medical School, Athens, Greece. The study protocol was approved by the Ethics Committee of Sotiria Chest Hospital (Approval No. 5024/ 25-05-2017) and all participants provided written informed consent in accordance with the Declaration of Helsinki. Participants were consecutively recruited from individuals attending the Allergy and Respiratory Departments between March 2017 and November 2019. A total of 80 participants presenting with rhinitis symptoms were enrolled and classified into two study groups. Patients with respiratory allergy were defined by a compatible clinical history together with evidence of sensitization demonstrated by at least one positive SPT and/or sIgE result. The comparison group consisted of symptomatic patients recruited from the same clinical setting who had rhinitis symptoms but negative conventional allergy testing (SPTs and sIgE). Because SPT and sIgE contributed to participant classification, these tests were not considered independent reference standards. Instead, they were used as established comparator diagnostic modalities against which the performance of NAPT and CAPT was evaluated. Consequently, the reported sensitivity estimates should be interpreted as measures of agreement with established diagnostic methods rather than validation against an independent gold standard. Spirometry was performed in all participants to assess pulmonary function and document the presence of concomitant asthma [32]. Spirometric findings were not used for participant allocation, diagnostic classification, or outcome assessment.

SPTs were carried out according to EAACI recommendations using standardized extracts [33]. The allergens tested were Cypressus arizonica, Olea europaea, mixed grasses, Phleum pratense, Parietaria judaica, Artemisia vulgaris, Alternaria alternata, Dermatophagoides pteronyssinus, and Dermatophagoides farinae, along with a positive control (histamine 10 mg/mL) and a negative control. Patients with respiratory allergy were defined as having at least one positive SPT reaction (wheal diameter ≥3 mm) and/or a positive specific IgE result (cut-off >0.35 kU/L). CRD was also included as an advanced technique enabling the identification of the patient’s molecular sensitization profile, allowing differentiation between primary sensitization and cross-reactivity, particularly in cases of multiple allergen sensitizations. The use of molecular diagnostics represents a precision medicine approach that may facilitate the optimal selection of allergens for specific immunotherapy [34-36].

Subsequently, specific nasal and conjunctival provocation tests to the relevant inhalant allergens were performed. NAPT and CAPT were not undertaken in all participants but were performed selectively according to clinical indication, sensitization profile, allergen relevance, and safety recommendations. The procedure for NAPT and CAPT involved standardized allergen extracts with a biological potency of 30 HEP/mL for Olea europaea, grass group, Phleum pratense, Parietaria judaica, Alternaria alternata, Cypressus arizonica, and Artemisia vulgaris, and 100 HEP/mL for house dust mites (HDMs), namely Dermatophagoides pteronyssinus and Dermatophagoides farinae. Provocation tests were performed during off-peak pollen periods for patients sensitized to seasonal allergens, and during periods of minimal symptom activity for those with perennial allergies [37,38]. All procedures were conducted in accordance with established safety recommendations, taking into account absolute and relative contraindications [17,39,40].

Prior to NAPT, a control challenge using normal saline or diluent was performed to exclude nonspecific nasal hyperreactivity. Thereafter, allergen provocation was carried out using increasing concentrations of the allergen extract at 15-minute intervals, up to the undiluted extract, until a positive clinical response was observed. The evaluation of NAPT included both subjective and objective parameters. Subjective assessment comprised nasal symptoms (rhinorrhea, nasal obstruction, sneezing, nasal pruritus) and associated ocular symptoms during both immediate and late phases, quantified using the Linder scoring scale [41]. Objective assessment included measurement of peak nasal inspiratory flow (PNIF) using the Clement Clarke International In-Check Nasal Meter [42,43]. PNIF is recommended by the AAAAI as part of standardized NAPT protocols when combined with symptom scores [44]. A positive NAPT result was determined according to established guideline-based criteria incorporating both symptom scores and objective nasal airflow measurements, interpreted in the context of the overall clinical response.

The CAPT procedure involved the instillation of two drops of diluent into the inferior bulbar conjunctiva, followed by administration of standardized allergen extracts at increasing concentrations at 15-minute intervals, up to the undiluted extract, until a positive response was observed. Clinical evaluation was based on the total ocular symptom score (TOSS), as recommended in CAPT guidelines, incorporating ocular pruritus, redness, lacrimation, chemosis, and eyelid edema [45]. A positive CAPT result was determined according to established guideline-based clinical criteria based on the development of characteristic ocular symptoms and signs following allergen exposure.

Blinding was not performed because allergen provocation procedures required direct clinical assessment of participant responses during testing.

Quantitative variables were expressed as mean (standard deviation) or median (interquartile range), while categorical variables were presented as absolute and relative frequencies. Group comparisons were performed using chi-square or Fisher’s exact test for categorical variables, and Student’s t-test or Mann-Whitney U test for continuous variables, as appropriate. The discriminative ability of selected provocation tests was initially assessed using receiver operating characteristic (ROC) curve analysis, with calculation of the area under the curve (AUC) and corresponding 95% confidence intervals. Diagnostic indices including sensitivity, specificity, positive predictive value, negative predictive value, and overall accuracy were also calculated. Statistical significance was defined as p<0.05 (two-tailed). These initial analyses were performed using IBM SPSS Statistics for Windows, Version 27 (Released 2019; IBM Corp., Armonk, New York, United States).

To further enhance the analytical approach, additional advanced statistical analyses were subsequently conducted. Multivariable logistic regression models were constructed to identify independent predictors of positive NAPT and CAPT responses, including age, total IgE (log-transformed), presence of asthma, and polysensitization status. Model discrimination was evaluated using ROC curve analysis with bootstrap-derived 95% confidence intervals for the AUC. Predictor variables were selected a priori based on clinical relevance and previous literature and were entered simultaneously into the regression models. Analyses were performed using complete-case data, with participants excluded only when required variables were unavailable. Multicollinearity was assessed using variance inflation factors, and no evidence of problematic multicollinearity was identified. Bootstrap confidence intervals were estimated using 2000 resamples. Calibration was assessed using locally weighted scatterplot smoothing (LOWESS) with bootstrap-derived confidence bands, and model fit was further evaluated using the Hosmer-Lemeshow goodness-of-fit test.

Agreement between diagnostic modalities (SPT, sIgE, CRD, NAPT, and CAPT) was evaluated using Cohen’s kappa coefficient. Subgroup analyses were performed to explore differences according to sensitization pattern (mono- versus polysensitized), type of allergen exposure (seasonal versus perennial), and presence of asthma. In addition, DCA was conducted to assess the potential clinical utility of the predictive models across a range of clinically relevant threshold probabilities, comparing net benefit between the prediction model, treat-all, and treat-none strategies. Confidence intervals for net benefit estimates were obtained using bootstrap resampling (2000 iterations).

**Figure 1:**
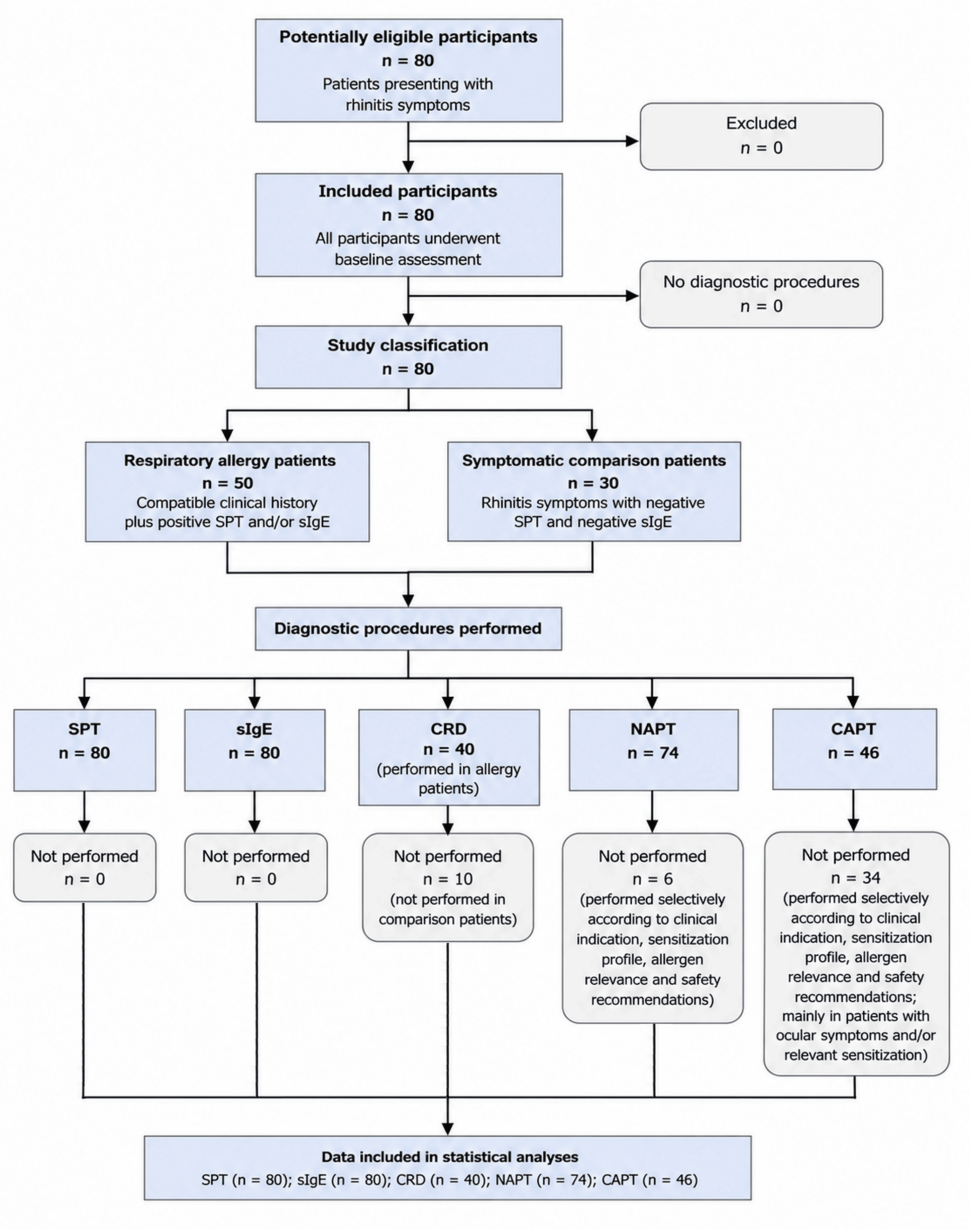
STARD-style participant flow diagram. The diagram summarizes participant enrollment, study classification, and completion of diagnostic procedures. Skin prick testing (SPT) and serum-specific IgE (sIgE) testing were performed in all participants. Component-resolved diagnostics (CRD) were performed in patients with respiratory allergy to further characterize molecular sensitization profiles. Nasal allergen provocation testing (NAPT) and conjunctival allergen provocation testing (CAPT) were performed selectively according to clinical indication, sensitization profile, allergen relevance, and safety recommendations. CAPT was mainly performed in selected patients with ocular symptoms and/or clinically relevant sensitization supporting conjunctival testing.

All advanced statistical analyses and figure generation were performed using Python (version 3.11.9), employing validated scientific libraries including pandas for data handling, NumPy for numerical computations, SciPy for statistical testing, scikit-learn for predictive modeling and ROC analysis, statsmodels for regression modeling and calibration methods, and matplotlib for figure generation. All analyses were conducted in accordance with TRIPOD-AI and the Standards for Reporting Diagnostic Accuracy Studies (STARD) recommendations [46]. A STARD-style participant flow diagram summarizing participant allocation and completion of diagnostic procedures is presented in Figure 1.

## Results

Participant flow through the study and completion of diagnostic procedures are summarized in Figure 1. The sample consisted of 80 participants, of whom 50/80 (62.5%) were patients with respiratory allergy. Their characteristics are presented in Table 1. The mean age of the total sample was 41.7 years (SD=14.2), and most participants were female (52/80, 65.0%). A personal history of atopy was present in all patients with respiratory allergy (50/50, 100.0%), while 45/80 (56.3%) reported a family history of atopy and 59/80 (73.8%) were non-smokers.

**Table 1:** Sample characteristics, in total sample and by group. +Pearson's chi-square test; ++Fisher's exact test; ‡Student's t-test; ‡‡Mann-Whitney test; – = not applicable or statistical comparison not performed; AD: atopic dermatitis

		Total sample (n=80; 100%)		Group				Ρ
				Control (n=30; 37.5%)		Patients with respiratory allergy (n=50; 62.5%)		
		Mean	SD	Mean	SD	Mean	SD	
Age (years)		41.7	14.2	45.9	14.9	39.2	13.3	0.039‡
		n	%	n	%	n	%	P
Gender	Male	28	35.0	9	30.0	19	38.0	0.468+
	Female	52	65.0	21	70.0	31	62.0	
Personal history of atopy		80	100.0	30	100.0	50	100.0	-
Rhinitis		80	100.0	30	100.0	50	100.0	-
Conjunctivitis		32	40.0	3	10.0	29	58.0	<0.001+
Asthma		35	43.8	5	16.7	30	60.0	<0.001+
Food allergy		2	2.5	0	0.0	2	4.0	0.525++
Drug allergy		6	7.5	2	6.7	4	8.0	>0.999++
Urticaria		7	8.8	2	6.7	5	10.0	0.706++
Eczema/AD		3	3.8	2	6.7	1	2.0	0.553++
Family atopic history		45	56.3	10	33.3	35	70.0	0.001+
Smoking history	Current smoker	7	8.8	3	10.0	4	8.0	0.411++
	Non-smoker	59	73.8	20	66.7	39	78.0	
	Occasional smoker	4	5.0	1	3.3	3	6.0	
	Ex-smoker	10	12.5	6	20.0	4	8.0	
Laboratory parameters		Median	IQR	Median	IQR	Median	IQR	P
Εο (cells/μl)		199	105 ─ 355	189	130 ─ 370	200	100 ─ 340	0.450‡‡
IgE total (ΙU/mL)		53.7	24.8 ─ 156.3	45	12.2 ─ 64	84.2	29.7 ─ 202	0.006‡‡

Compared with symptomatic patients with negative conventional allergy testing, patients with respiratory allergy were significantly younger (39.2±13.3 vs 45.9±14.9 years; p=0.039), more frequently reported conjunctivitis (29/50, 58.0% vs 3/30, 10.0%; p<0.001), asthma (30/50, 60.0% vs 5/30, 16.7%; p<0.001), and a family history of atopy (35/50, 70.0% vs 10/30, 33.3%; p=0.001). Total IgE levels were also significantly higher in patients (p=0.006). No statistically significant differences were observed between groups in smoking status, eosinophil counts, or the prevalence of food allergy, drug allergy, urticaria, or eczema (p>0.05).

Not all diagnostic procedures were performed in all participants. Molecular diagnostics (CRD) were performed only in patients with evidence of sensitization on conventional testing in order to further characterize molecular sensitization profiles. Likewise, NAPT and CAPT were performed only in participants with clinically relevant sensitization for the allergen under investigation and when testing was considered clinically indicated according to the study protocol and safety recommendations. Consequently, the denominators reported for CRD, NAPT, and CAPT differ from the total study population and reflect the number of participants who underwent each specific procedure.

Among the total sample, 50/80 (62.5%) had a positive SPT, 40/80 (50.0%) had a positive specific IgE result, 40/50 (80.0%) had a positive molecular diagnostic result, 33/74 (44.6%) had a positive NAPT, and 42/46 (91.3%) had a positive CAPT (Table 2). In the patient group, positivity rates were 50/50 (100.0%) for SPT, 40/50 (80.0%) for sIgE and CRD, whereas no participant in the comparison group demonstrated positive SPT or sIgE results. Because SPT and sIgE contributed to participant classification, these findings should be interpreted as descriptive characteristics of the study population rather than independent validation measures.

**Table 2:** Positive results in SPTs, laboratory tests, molecular diagnostics, nasal provocation tests, and conjunctival provocation tests. Percentages are reported using the number of participants who underwent the corresponding diagnostic procedure as the denominator. Molecular diagnostics and provocation tests were performed only in selected participants according to sensitization profile and clinical indication. *p<0.05; **p<0.01; ***p<0.001 for comparison between patients and controls; –: not applicable (molecular and provocation test results are reported only for relevant sensitized/allergic subjects); SPT: skin prick test

Variable	Total sample		Group			
			Control		Patients with respiratory allergy	
	n	%	n	%	n	%
Positive SPTs	50	62.5	0	0.0	50	100.0***
Cypressus arizonica	13	16.3	0	0.0	13	26.0**
Olea europaea	15	18.8	0	0.0	15	30.0***
Grasses group	5	6.3	0	0.0	5	10.0
Timothy grass	5	6.3	0	0.0	5	10.0
Parietaria judaica	22	27.5	0	0.0	22	44.0***
Artemisia vulgaris	5	6.3	0	0.0	5	10.0
Alternaria alternata	4	5.0	0	0.0	4	8.0
Dermatophagoides farinae	17	21.3	0	0.0	17	34.0**
Dermatophagoides pteronyssinus	19	23.8	0	0.0	19	38.0**
Positive laboratory test	40	50.0	0	0.0	40	80.0***
Cypressus arizonica t222	5	6.3	0	0.0	5	10.0
Olea europaea t9	7	8.8	0	0.0	7	14.0*
Grasses group gx1	3	3.8	0	0.0	3	6.0
Timothy grass g6	3	3.8	0	0.0	3	6.0
Parietaria judaica w21	20	25.0	0	0.0	20	40.0***
Artemisia vulgaris w6	4	5.0	0	0.0	4	8.0
Alternaria alternata m6	3	3.8	0	0.0	3	6.0
Dermatophagoides farinae d2	16	20.0	0	0.0	16	32.0***
Dermatophagoides pteronyssinus d1	15	18.8	0	0.0	15	30.0***
Positive molecular laboratory test	40	80.0	-	-	40	80.0
Cypressus arizonica t226 (n=13)	8	61.5	-	-	8	61.5
Olea europaea t224 (n=15)	12	80.0	-	-	12	80.0
Timothy grass g205 (n=5)	3	60.0	-	-	3	60.0
Parietaria judaica w211 (n=22)	19	86.4	-	-	19	86.4
Artemisia vulgaris w231 (n=5)	2	40.0	-	-	2	40.0
Alternaria alternata m229 (n=4)	4	100.0	-	-	4	100.0
Dermatophagoides pteronyssinus d202 (n=22)	7	31.8	-	-	7	31.8
Dermatophagoides pteronyssinus d203 (n=22)	12	54.5	-	-	12	54.5
Dermatophagoides pteronyssinus d205 (n=22)	1	4.5	-	-	1	4.5
Nasal provocation tests (n=74)	33	44.6	0	0.0	33	75.0***
Olea europaea (n=45)	11	24.4	0	0.0	11	73.3***
Grasses group (n=5)	2	40.0	-	-	2	40.0
Timothy grass (n=5)	2	40.0	-	-	2	40.0
Parietaria judaica (n=22)	19	86.4	-	-	19	86.4
Alternaria alternata (n=4)	3	75.0	-	-	3	75.0
Dermatophagoides pteronyssinus (n=52)	11	21.2	0	0.0	11	50.0***
Dermatophagoides farinae (n=21)	9	42.9	-	-	9	42.9
Conjunctival provocation tests (n=46)	42	91.3	-	-	42	91.3
Cypressus arizonica (n=13)	8	61.5	-	-	8	61.5
Timothy grass (n=5)	3	60.0	-	-	3	60.0
Parietaria judaica (n=22)	22	100.0	-	-	22	100.0
Artemisia vulgaris (n=5)	3	60.0	-	-	3	60.0
Dermatophagoides pteronyssinus (n=22)	14	63.6	-	-	14	63.6
Dermatophagoides farinae (n=21)	13	61.9	-	-	13	61.9

The results presented in Table 2 include both participant-level and allergen-level analyses. Participant-level analyses refer to the proportion of subjects with at least one positive result for a given diagnostic modality, whereas allergen-level analyses describe positivity rates for specific allergens among the participants who underwent testing for that allergen. Molecular diagnostics (CRD) were performed in sensitized patients to further characterize molecular sensitization profiles. NAPT and CAPT were performed only when clinically indicated for relevant allergens according to the study protocol and safety recommendations. Consequently, the denominators differ across diagnostic procedures and allergen-specific analyses and reflect the number of participants who underwent each respective test.

Regarding allergen-specific sensitization, SPT positivity was most frequently observed for Parietaria judaica (22/80, 27.5%) and Dermatophagoides pteronyssinus (19/80, 23.8%), while laboratory testing most commonly identified sensitization to Parietaria judaica (20/80, 25.0%) and Dermatophagoides farinae (16/80, 20.0%). Molecular diagnostics demonstrated the highest positivity rates for Alternaria alternata (4/4, 100.0%) and Parietaria judaica (19/22, 86.4%). In nasal provocation testing, the most frequent positive responses were observed for Parietaria judaica (19/22, 86.4%) and Alternaria alternata (3/4, 75.0%). In conjunctival provocation testing, all participants tested for Parietaria judaica demonstrated a positive response (22/22, 100.0%). However, because CAPT was performed in selected subjects and no independent reference standard was available, these findings should be interpreted cautiously.

Detailed allergen-specific analyses demonstrated variability in NAPT sensitivity across allergens. The overall sensitivity of NAPT was 33/44 (75.0%) compared with SPTs, 29/37 (78.4%) compared with sIgE, and 31/36 (86.1%) compared with molecular diagnostics. Sensitivity varied considerably across allergens, ranging from 2/5 (40.0%) for grasses and Phleum pratense to 19/22 (86.4%) for Parietaria judaica for SPT comparisons, and from 9/16 (56.3%) for Dermatophagoides farinae to 7/7 (100.0%) for Olea europaea for sIgE comparisons. Similar variability was observed when compared with molecular diagnostics.

CAPT demonstrated consistently high sensitivity across allergens and diagnostic methods. It showed higher sensitivity than NAPT, with overall sensitivity of 42/46 (91.3%) compared with SPTs, 37/39 (94.9%) compared with sIgE, and 36/38 (94.7%) compared with molecular diagnostics. Allergen-specific sensitivity ranged from 3/5 (60.0%) to 22/22 (100.0%), with consistently high performance for Parietaria judaica across all reference methods.

**Figure 2:**
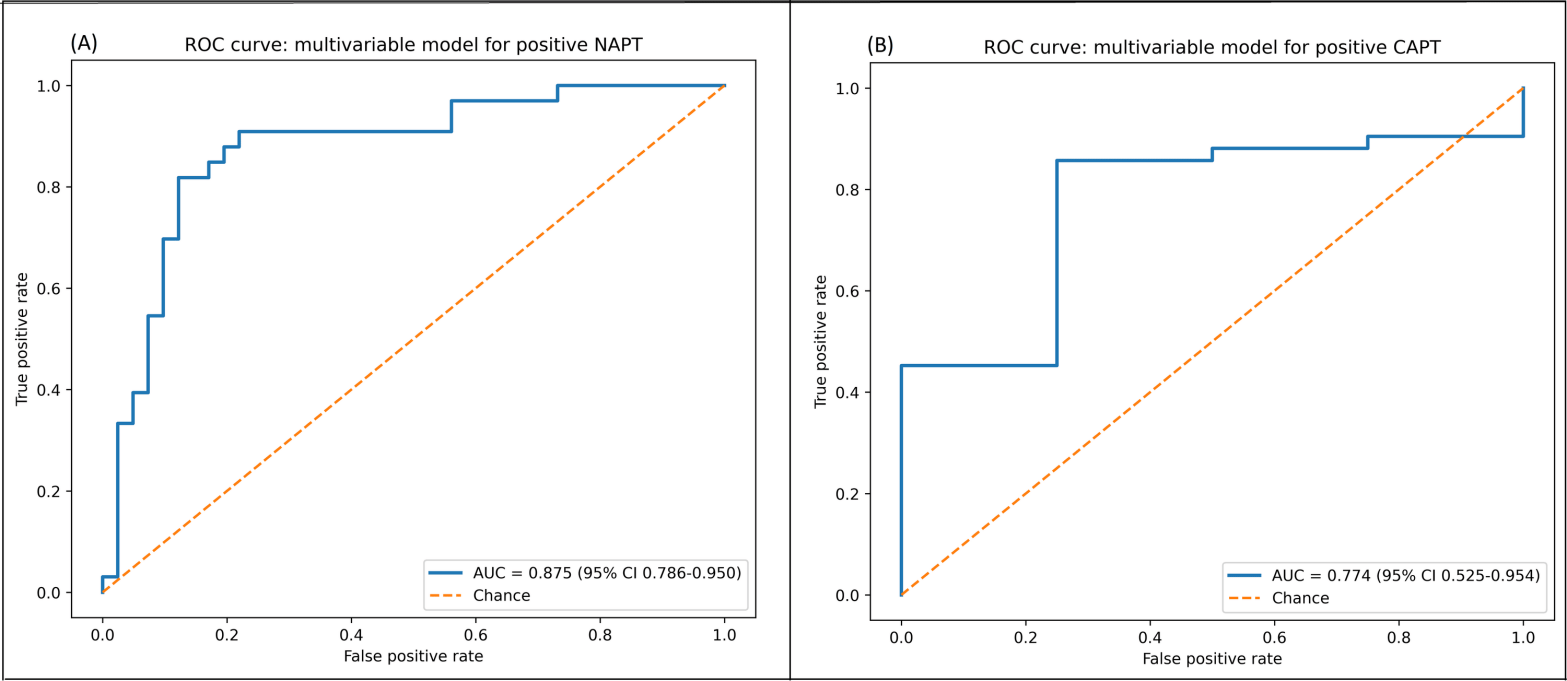
ROC analysis of multivariable models predicting positive nasal and conjunctival allergen provocation test responses. (A) ROC curve of the multivariable model for positive NAPT (n=74; 33 positive and 41 negative outcomes), demonstrating an AUC of 0.875 (95% bootstrap CI: 0.786–0.950). (B) ROC curve of the multivariable model for positive CAPT (n=46; 42 positive and 4 negative outcomes), demonstrating an AUC of 0.774 (95% bootstrap CI: 0.525–0.954). Both models included age, log-transformed total IgE, asthma, and polysensitization status as predictor variables. ROC performance and confidence intervals were estimated using bootstrap resampling (2000 iterations). ROC: receiver operating characteristic; AUC: area under the curve; CI: confidence interval; NAPT: nasal allergen provocation testing; CAPT: conjunctival allergen provocation testing; IgE: immunoglobulin E. The figure was generated directly from the study dataset using author-developed Python scripts (version 3.11.9) provided in the Appendices.

ROC curve analysis of the multivariable models demonstrated good discriminative ability for prediction of positive provocation test responses (Figures 2A, 2B). The model for positive NAPT showed an area under the curve (AUC) of 0.875 (95% bootstrap CI: 0.786-0.950), whereas the model for positive CAPT showed an AUC of 0.774 (95% bootstrap CI: 0.525-0.954). Overall diagnostic performance of the evaluated diagnostic methods is summarized in Table 3.

**Table 3:** Nasal provocation tests in Olea europaea and Dermatophagoides pteronyssinus by group. PPV: positive predictive value; NPV: negative predictive value

		Control group		Patients with respiratory allergy		Sensitivity (%)	Specificity (%)	PPV (%)	NPV (%)	Accuracy (%)
Nasal challenge		n	%	n	%					
Olea europaea	Negative	30	100.0	4	26.7	73.3	100	100	91.1	91.1
	Positive	0	0.0	11	73.3					
Dermatophagoides pteronyssinus	Negative	30	100.0	11	50.0	50	100	100	73.2	78.8
	Positive	0	0.0	11	50.0					

**Figure 3:**
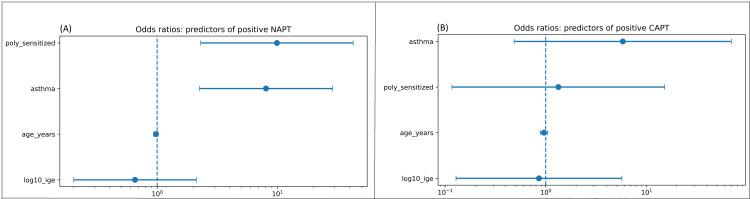
Multivariable predictors of positive nasal and conjunctival allergen provocation test responses. (A) ORs with 95% CIs for predictors of NAPT. Asthma and polysensitization were independently associated with positive NAPT responses, whereas age and total IgE were not significant predictors. (B) ORs with 95% CIs for predictors of positive CAPT. None of the evaluated predictors reached statistical significance in the CAPT model. Both multivariable logistic regression models included age, log-transformed total IgE, asthma, and polysensitization status as predictor variables. OR: odds ratio; CI: confidence interval; NAPT: nasal allergen provocation testing; CAPT: conjunctival allergen provocation testing; IgE: immunoglobulin E. The dashed vertical line indicates an OR of 1.0 (no association). Error bars represent 95% confidence intervals. The figure was generated directly from the study dataset using author-developed Python scripts (version 3.11.9) provided in the Appendices.

Multivariable logistic regression models were constructed using age, log-transformed total IgE, asthma, and polysensitization status as predictors. The NAPT model included 74 participants who underwent NAPT (33 positive and 41 negative outcomes), whereas the CAPT model included 46 participants who underwent CAPT (42 positive and 4 negative outcomes). In the NAPT model, asthma (OR=8.05, 95% CI: 2.25-28.73; p=0.001) and polysensitization (OR=9.92, 95% CI: 2.30-42.73; p=0.002) were independently associated with positive NAPT responses (Figure 3A), whereas age and total IgE were not significant predictors. In the CAPT model, none of the evaluated predictors reached statistical significance (Figure 3B), likely reflecting the substantial imbalance between positive and negative outcomes.

**Figure 4:**
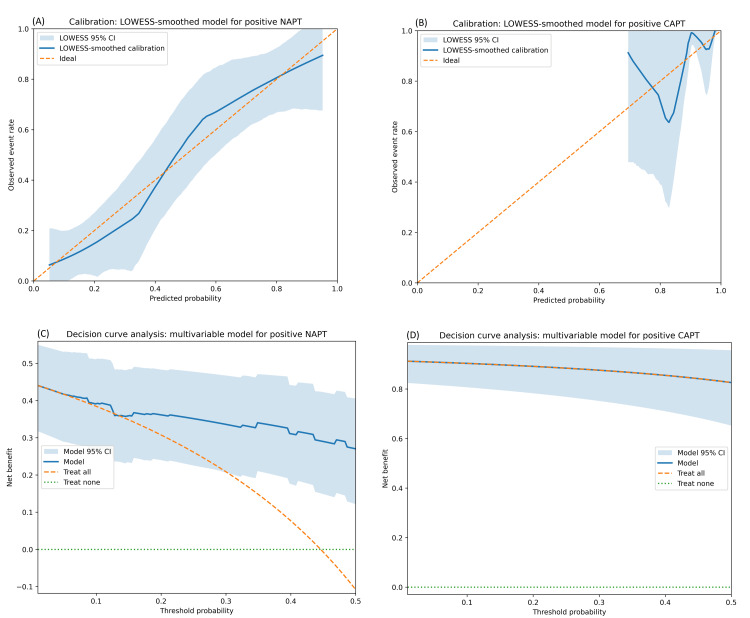
Calibration and clinical utility of multivariable models for nasal and conjunctival allergen provocation test responses. (A) Calibration plot for the multivariable model predicting positive NAPT using LOWESS smoothing with bootstrap-derived 95% CI. (B) Calibration plot for the multivariable model predicting positive CAPT. (C) Decision curve analysis for the multivariable model predicting positive NAPT. (D) Decision curve analysis for the multivariable model predicting positive CAPT. LOWESS: locally weighted scatterplot smoothing; CI: confidence interval; NAPT: nasal allergen provocation testing; CAPT: conjunctival allergen provocation testing; DCA: decision curve analysis. The figure was generated directly from the study dataset using author-developed Python scripts (version 3.11.9) provided in the Appendices.

Calibration analysis using LOWESS-smoothed curves demonstrated acceptable agreement between predicted and observed probabilities for the NAPT model, whereas calibration for the CAPT model was less stable. The apparent instability of the CAPT calibration curve likely reflects the high prevalence of positive responses and the limited number of negative outcomes available for model estimation (Figures 4A, 4B). Hosmer-Lemeshow testing did not indicate significant lack of fit for the NAPT model.

Agreement analysis demonstrated substantial agreement between SPT and sIgE (κ=0.750; n=80), fair-to-moderate agreement between sIgE and CRD (κ=0.375; n=50), and moderate agreement between CRD and NAPT (κ=0.533; n=44). Agreement between CRD and CAPT was lower (κ=0.246; n=46), indicating greater divergence between conjunctival provocation responses and sensitization-based diagnostic methods. These findings suggest that provocation tests may capture aspects of clinical reactivity that are not fully reflected by conventional sensitization-based assessments and support their complementary role in selected patients.

Subgroup analyses demonstrated higher positivity rates of provocation tests in polysensitized patients and in those with asthma, supporting their role in more complex clinical phenotypes. DCA further indicated that the NAPT-based model provided a higher net clinical benefit across a range of threshold probabilities compared with treat-all and treat-none strategies, supporting its potential utility in clinical decision-making (Figure 4C). In contrast, the CAPT-based model showed less consistent net benefit across threshold probabilities (Figure 4D).

**Figure 5:**
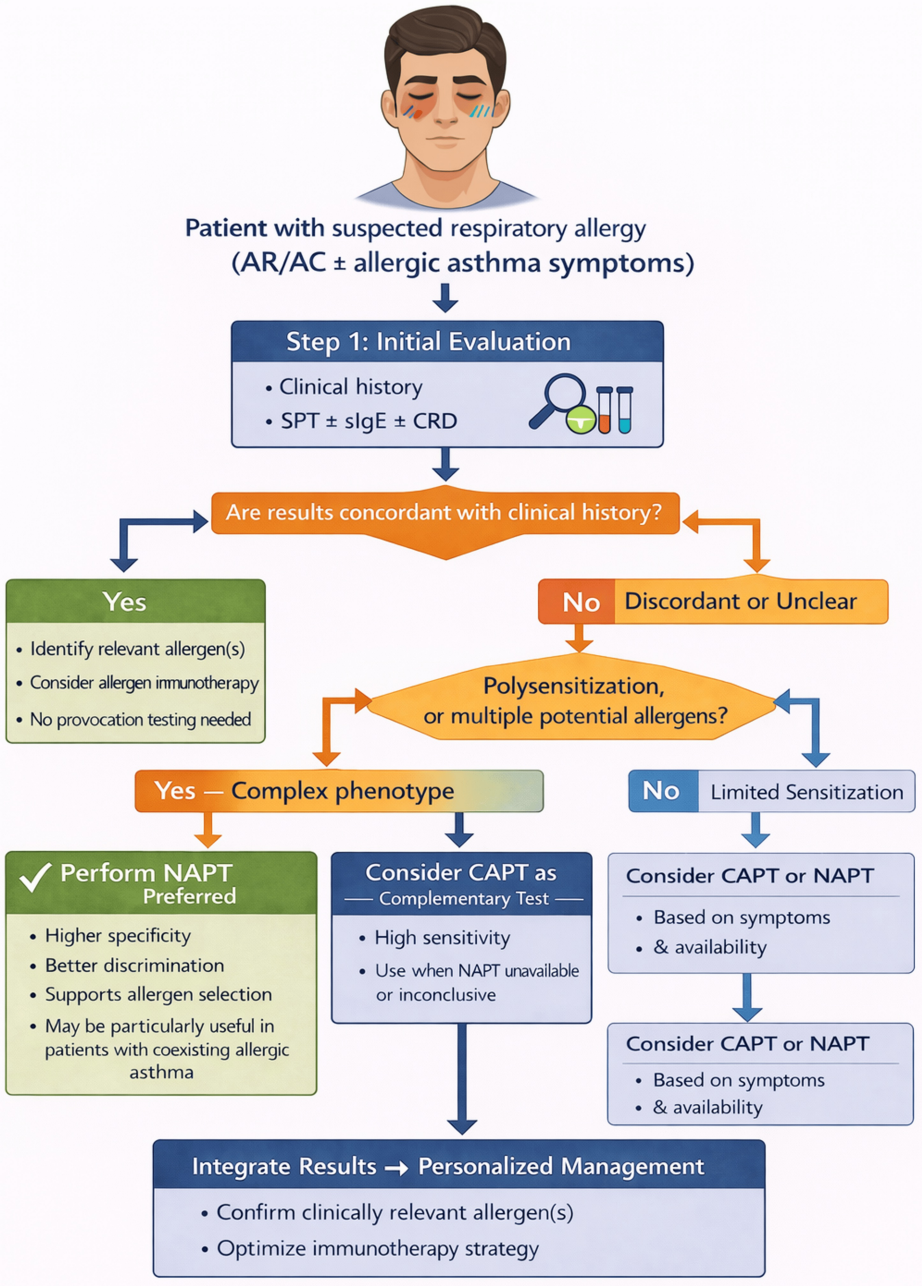
Proposed diagnostic algorithm for the integration of nasal and conjunctival allergen provocation tests in patients with suspected respiratory allergy. This algorithm illustrates a proposed approach for integrating NAPT and CAPT allergen provocation tests into the diagnostic workup of respiratory allergy. NAPT may be particularly useful in patients with polysensitization or discordant clinical and sensitization findings, due to its higher discriminative capacity. CAPT, while highly sensitive, may serve as a complementary tool. The algorithm is intended to support clinical decision-making and should be applied in the context of individual patient characteristics and local expertise. This figure was created using Canva (Canva Pty Ltd., Sydney, Australia). NAPT: nasal allergen provocation testing; CAPT: conjunctival allergen provocation testing; SPT: skin prick test; sIgE: serum-specific immunoglobulin E; CRD: component-resolved diagnostics; AR: allergic rhinitis; AC: allergic conjunctivitis.

Overall, these findings indicate that CAPT demonstrated higher positivity rates and sensitivity relative to the comparator diagnostic methods evaluated in this study. However, given the high prevalence of positive CAPT responses and the absence of an independent reference standard, these findings should be interpreted cautiously. In contrast, NAPT demonstrated greater discriminative performance, more stable calibration characteristics, and potentially greater clinical utility, particularly in complex or polysensitized patients. Detailed allergen-specific sensitivity analyses and agreement metrics supported the consistency and robustness of the main findings. To facilitate the translation of these findings into clinical practice, a structured diagnostic algorithm integrating nasal and conjunctival allergen provocation testing is proposed (Figure 5). This framework highlights the potential added value of NAPT in complex or polysensitized patients, while positioning CAPT as a complementary tool in selected cases. By incorporating provocation testing into routine diagnostic pathways, this approach may improve diagnostic precision and support more individualized treatment decisions, including optimization of allergen immunotherapy. Nevertheless, the proposed algorithm should be interpreted in the context of the study design and requires validation in larger multicenter populations before routine implementation.

## Discussion

The present study provides a comprehensive evaluation of nasal and conjunctival allergen provocation tests in comparison with conventional and molecular diagnostic approaches, extending beyond traditional measures of diagnostic accuracy. By incorporating advanced statistical modeling, including calibration and DCA, this study offers additional insight into the clinical utility of provocation testing in real-world settings. The findings highlight important differences between NAPT and CAPT, not only in terms of sensitivity and specificity but also in their capacity to inform clinical decision-making, particularly in patients with complex or polysensitized allergic phenotypes.

All participants in the present study reported a personal atopic history, and more than half had a familial atopic background, consistent with the well-established genetic predisposition underlying allergic respiratory diseases. Total IgE levels were significantly higher in patients with respiratory allergy, reinforcing its role as a biomarker of Th2-driven inflammation in conditions such as allergic rhinitis and asthma. Previous studies have demonstrated both systemic and local IgE production, including persistent nasal IgE synthesis even outside allergen exposure periods [47,48].

The distribution of sensitization to inhalant allergens in our cohort reflects regional epidemiological patterns. Sensitization to Dermatophagoides pteronyssinus and Parietaria judaica was particularly prevalent, in line with previous epidemiologic reports [49]. In contrast, sensitization to grass pollen appeared less frequent, highlighting the influence of geographical and environmental factors on allergen exposure and sensitization profiles.

Serum sIgE measurement demonstrated good concordance with SPTs, with an overall sensitivity of 80%, supporting its role as a reliable in vitro diagnostic method. Previous studies have reported similarly high sensitivity and specificity of sIgE assays, particularly for HDM allergens [50,51,52]. However, these results should be interpreted in conjunction with clinical history and complementary diagnostic modalities, as sensitization alone does not necessarily correspond to clinically relevant allergic disease.

CRD further enhanced the characterization of sensitization profiles, particularly in polysensitized patients. In the present study, CRD showed comparable sensitivity to conventional methods, consistent with previous reports demonstrating its high diagnostic accuracy [53,54]. Molecular diagnostics allow differentiation between primary sensitization and cross-reactivity, thereby improving patient stratification and potentially guiding allergen immunotherapy [53,55-58].

A central finding of the present study is the differential diagnostic performance of nasal and conjunctival allergen provocation tests. While both methods demonstrated high sensitivity, CAPT consistently exhibited higher sensitivity across all reference standards, exceeding 90% in most comparisons. These findings are in agreement with previous studies reporting high diagnostic performance of CAPT, particularly in ocular allergy and HDM sensitization [59-65]. CAPT has also been widely used in the evaluation of ocular allergic diseases, including vernal keratoconjunctivitis and IgE-mediated conjunctival inflammation [61-63]. However, the very high positivity rate observed for CAPT in our cohort may also reflect reduced specificity or limited discriminatory capacity in highly sensitized populations, particularly when used in isolation.

In contrast, NAPT demonstrated moderate but clinically meaningful sensitivity, with variability across allergens. This variability has been reported in previous studies and may reflect differences in allergen characteristics, exposure patterns, and methodological approaches [66-70]. Importantly, NAPT provides direct evidence of target organ reactivity, which may more closely reflect clinically meaningful disease compared with sensitization-based testing alone. This distinction is particularly relevant in polysensitized patients, in whom conventional diagnostic methods may not reliably identify the clinically dominant allergen [15,16].

The incorporation of advanced statistical modeling in the present study provides additional insight into the clinical utility of provocation testing. Multivariable logistic regression identified polysensitization and asthma as the strongest predictors of positive NAPT responses, supporting the concept that provocation testing is particularly informative in patients with more complex or severe allergic phenotypes. In contrast, age and total IgE demonstrated weaker associations, suggesting that clinical reactivity is not solely determined by systemic immunological markers.

Furthermore, ROC curve analysis demonstrated good discriminative performance of NAPT, particularly for Olea europaea, with high specificity and overall diagnostic accuracy. Calibration analysis indicated satisfactory agreement between predicted and observed probabilities for the NAPT model, whereas CAPT models showed less stable calibration, likely reflecting the high prevalence of positive responses. DCA further demonstrated that the NAPT-based model provided greater net clinical benefit across clinically relevant threshold probabilities, supporting its potential role in guiding clinical decision-making. In contrast, the CAPT model showed limited incremental net benefit beyond a treat-all strategy, suggesting that its very high sensitivity may be accompanied by reduced ability to meaningfully stratify patients according to risk.

Agreement analysis between diagnostic modalities revealed that provocation tests capture dimensions of allergic disease not fully reflected by sensitization-based methods. The moderate agreement between CRD and NAPT, and the lower agreement with CAPT, further support the concept that functional testing provides complementary information regarding clinical reactivity. These findings are particularly relevant in real-world settings, where discrepancies between clinical history and sensitization results are frequently encountered.

The safety profile of provocation testing in the present study was consistent with previous reports. No severe adverse events were observed, and no asthma exacerbations occurred during NAPT, supporting its use in controlled clinical settings with appropriate patient selection and monitoring [59,71-74]. Nasal allergen provocation may also induce lower airway responses, supporting the concept of united airway disease [75,76].

From a clinical perspective, the present findings support a more integrated diagnostic approach in patients with suspected respiratory allergy, particularly in cases characterized by polysensitization or discordance between clinical history and sensitization-based testing. Nasal allergen provocation testing appears to provide added value by identifying symptom-relevant reactivity and refining allergen attribution, which may be especially useful when selecting candidates for allergen immunotherapy or determining the most appropriate allergen composition. In contrast, although conjunctival provocation testing demonstrates high sensitivity, its lower discriminative capacity may limit its utility as a stand-alone tool in complex cases. Incorporating both methods within a structured diagnostic framework may enhance overall diagnostic precision and clinical decision-making. In this context, further research should evaluate the cost-effectiveness of integrating provocation testing into routine clinical pathways, particularly in relation to optimizing resource utilization and improving treatment selection.

Several limitations should be acknowledged. First, participant classification was based in part on conventional allergy testing (SPT and/or sIgE) combined with clinical history. Consequently, incorporation bias cannot be completely excluded when comparisons are made between provocation tests and sensitization-based diagnostic methods. The present study was designed to evaluate concordance and comparative diagnostic performance among available diagnostic approaches rather than to validate any test against an independent gold standard. Second, the study was conducted in a single center, which may limit the generalizability of the findings. However, the study population reflects real-world clinical practice in a Mediterranean setting, where polysensitization is common. Third, the sample size was relatively small for certain allergens, which may have reduced statistical power in subgroup analyses, although the consistency of effect estimates across analyses supports the robustness of the findings. Fourth, provocation outcomes were not assessed in a blinded manner, which may introduce observer bias; however, standardized protocols and objective measurements were applied to mitigate this limitation. Fifth, long-term clinical outcomes, including response to allergen immunotherapy, were not evaluated and warrant further investigation. Moreover, external validation was not feasible due to the lack of comparable datasets incorporating provocation testing; however, internal validation using bootstrap resampling demonstrated stable model performance. Finally, the CAPT predictive model should be interpreted cautiously because only four CAPT-negative outcomes were available, resulting in substantial outcome imbalance and potentially unstable regression coefficient estimates and performance metrics.

## Conclusions

Both nasal and conjunctival allergen provocation tests demonstrated high positivity rates in patients with respiratory allergy, although their diagnostic roles differed. CAPT showed higher positivity rates across the evaluated diagnostic methods, whereas NAPT demonstrated greater discriminative performance and more stable calibration characteristics within the present dataset.

These findings suggest that provocation testing may provide complementary information to conventional sensitization-based diagnostic methods, particularly in patients with polysensitization or discordant clinical and laboratory findings. However, the results should be interpreted in light of the relatively small sample size, selective application of diagnostic procedures, and absence of an independent reference standard. NAPT and CAPT may therefore serve as complementary diagnostic tools in selected patients, but further multicenter studies with larger populations and outcome-based validation are needed to confirm their clinical utility and define their role in routine allergy practice.

## Figures and Tables

**Figure 6:**
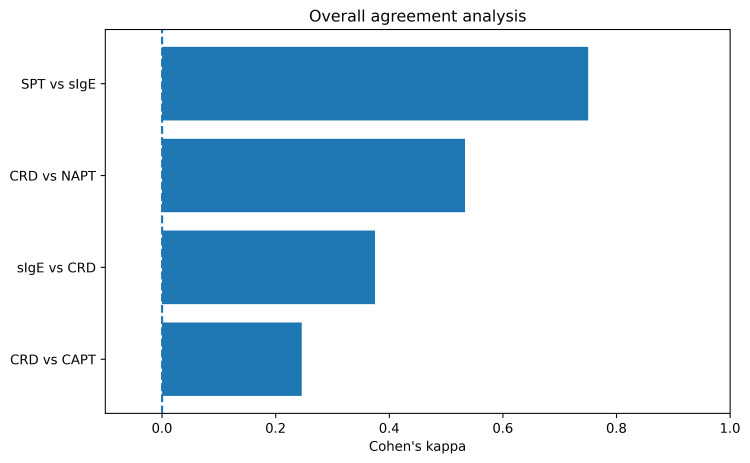
Agreement between diagnostic modalities in respiratory allergy assessed by Cohen’s kappa coefficient. Agreement analysis demonstrated variable concordance between sensitization-based diagnostic methods (SPT, sIgE, and CRD) and provocation-based methods (NAPT and CAPT). Higher agreement was observed between SPT and sIgE, reflecting their common role in identifying allergen sensitization. In contrast, agreement between conventional diagnostic methods and provocation tests was lower, suggesting that provocation testing captures clinically relevant allergic reactivity not fully reflected by sensitization status alone. These findings support the complementary role of NAPT and CAPT, particularly in polysensitized patients and in cases where clinical history and sensitization results are discordant. SPT: skin prick test; sIgE: serum-specific immunoglobulin E; CRD: component-resolved diagnostics; NAPT: nasal allergen provocation testing; CAPT: conjunctival allergen provocation testing.

## Appendices

Appendix 1. Detailed statistical analysis and reproducibility framework

**Table 4:** Detailed statistical analysis and reproducibility framework This table summarizes the advanced statistical methods, model development procedures, validation strategy, and reproducibility framework applied in the present study. All advanced analyses were performed using Python version 3.11.9, while primary statistical analyses were conducted using SPSS version 27.0. Internal validation was performed using 2,000 bootstrap resamples. AUC: area under the curve; CAPT: conjunctival allergen provocation test; CI: confidence interval; CRD: component-resolved diagnostics; DCA: decision curve analysis; LOWESS: locally weighted scatterplot smoothing; NAPT: nasal allergen provocation testing; ORs: odds ratios; ROC: receiver operating characteristic; sIgE: serum-specific immunoglobulin E; SPT: skin prick test.

Domain	Methodological details
Study reporting framework	Statistical analyses and model reporting were conducted according to TRIPOD-AI principles and the Standards for Reporting Diagnostic Accuracy Studies (STARD).
Primary statistical software	Initial descriptive and inferential analyses were performed using SPSS version 27.0.
Advanced analytical software	Advanced modeling, validation procedures, agreement analyses, and figure generation were performed using Python version 3.11.9.
Data quality assessment	Prior to analysis, the dataset was evaluated for completeness, consistency, plausibility, and coding accuracy. Continuous variables were examined for distributional characteristics and transformed where appropriate. Categorical variables were harmonized to ensure consistent encoding across analyses.
Outlier management	Outliers were investigated and retained when clinically plausible.
Missing data	A complete-case approach was used because missing data were minimal and considered unlikely to influence model estimates.
Primary outcomes	Binary outcomes were defined as the presence of at least one positive NAPT response and at least one positive CAPT response.
Regression modeling	Multivariable logistic regression models were developed to identify factors associated with positive NAPT and CAPT responses. Results were expressed as ORs with corresponding 95% CIs.
Predictor variables	Age, total serum IgE, asthma status, and sensitization profile (monosensitized versus polysensitized) were included in all models based on clinical relevance and previous evidence.
Definition of polysensitization	Sensitization to two or more allergens identified by SPT, sIgE, or CRD.
Variable transformation	Total IgE demonstrated right-skewness and was therefore log10-transformed before model fitting to improve model stability and interpretability.
Overfitting prevention	The number of predictors was selected to maintain an adequate events-per-variable ratio and reduce the risk of model overfitting.
Multicollinearity assessment	Variance inflation factors (VIFs) were calculated. No significant multicollinearity was detected among predictors.
Discrimination assessment	ROC curve analysis was performed. AUC values and 95% CIs were estimated using bootstrap resampling (2,000 iterations).
Calibration assessment	Calibration was evaluated using LOWESS calibration curves with bootstrap-derived confidence bands. Model fit was additionally assessed using the Hosmer-Lemeshow goodness-of-fit test.
Clinical utility assessment	DCA was performed across threshold probabilities ranging from 0.01 to 0.50. Net benefit was compared between model-based, treat-all, and treat-none strategies.
Internal validation	Bootstrap resampling with 2,000 iterations was used to estimate optimism-corrected performance measures for discrimination, calibration, and clinical utility.
Agreement analysis	Agreement between SPT, sIgE, CRD, NAPT, and CAPT was assessed using Cohen’s kappa coefficient. Interpretation thresholds were: ≤0.20 poor; 0.21–0.40 fair; 0.41–0.60 moderate; 0.61–0.80 substantial; >0.80 excellent agreement.
Subgroup analyses	Prespecified subgroup analyses were performed according to sensitization pattern (monosensitized versus polysensitized), allergen exposure type (seasonal versus perennial), and asthma status.
Scientific libraries	pandas and NumPy were used for data management and numerical operations; SciPy for statistical testing; scikit-learn for ROC and predictive analyses; statsmodels for regression and calibration procedures; and matplotlib for figure generation.
Reproducibility resources	All analytical scripts used to generate the reported results, figures, and supplementary analyses are provided as appendices and may be executed using Python version 3.11.9 and the listed scientific libraries.

Appendix 2. Diagnostic performance of provocation tests compared with conventional and molecular diagnostic methods

**Table 5:** Sensitivity and false-negative rates of nasal allergen provocation testing (NAPT) compared with skin prick testing, serum-specific IgE, and component-resolved diagnostics. Sensitivity was calculated as the proportion of positive NAPT results among individuals with positive reference diagnostic tests. False-negative rates represent the proportion of patients with positive reference tests but negative NAPT results. SPT: Skin prick test; IgE: immunoglobulin E

	Nasal provocation tests			
	False negative		Sensitivity	
	n	%	n	%
Positive SPTs in:				
All allergens (n=44)	11	25.0	33	75.0
Olea europaea (n=15)	4	26.7	11	73.3
Grasses group (n=5)	3	60.0	2	40.0
Timothy grass (n=5)	3	60.0	2	40.0
Parietaria judaica (n=22)	3	13.6	19	86.4
Alternaria alternata (n=4)	1	25.0	3	75.0
Dermatophagoides farinae (n=17)	9	52.9	8	47.1
Dermatophagoides pteronyssinus (n=19)	9	47.4	10	52.6
Positive laboratory test in:				
All allergens (n=37)	8	21.6	29	78.4
Olea europaea t9 (n=7)	0	0.0	7	100.0
Grasses group gx1 (n=3)	1	33.3	2	66.7
Timothy grass g6 (n=3)	1	33.3	2	66.7
Parietaria judaica w21 (n=20)	2	10.0	18	90.0
Artemisia alternata w6 (n=3)	1	33.3	2	66.7
Dermatophagoides farinae d2 (n=16)	7	43.8	9	56.3
Dermatophagoides pteronyssinus d1 (n=15)	4	26.7	11	73.3
Positive molecular laboratory test in:				
All allergens (n=36)	5	13.9	31	86.1
Olea europaea t224 (n=12)	2	16.7	10	83.3
Timothy grass g205 (n=3)	1	33.3	2	66.7
Parietaria judaica w211 (n=19)	1	5.3	18	94.7
Alternaria alternata m229 (n=4)	1	25.0	3	75.0
Dermatophagoides pteronyssinus d202 (n=7)	2	28.6	5	71.4
Dermatophagoides pteronyssinus d203 (n=12)	2	16.7	10	83.3
Dermatophagoides pteronyssinus d205 (n=1)	1	100.0	0	0.0

**Table 6:** Sensitivity and false-negative rates of conjunctival allergen provocation testing (CAPT) compared with skin prick testing, serum-specific IgE, and component-resolved diagnostics. Sensitivity was calculated as the proportion of positive CAPT results among individuals with positive reference diagnostic tests. False-negative rates represent the proportion of patients with positive reference tests but negative CAPT results. IgE: Immunoglobulin E

	Conjunctival provocation tests			
	False negative		Sensitivity	
	n	%	n	%
Positive SPTs in:				
All allergens (n=46)	4	8.7	42	91.3
Cypressus arizonica (n=13)	5	38.5	8	61.5
Timothy grass (n=5)	2	40.0	3	60.0
Parietaria judaica (n=22)	0	0.0	22	100.0
Artemisia vulgaris(n=5)	2	40.0	3	60.0
Dermatophagoides farinae (n=17)	6	35.3	11	64.7
Dermatophagoides pteronyssinus (n=19)	5	26.3	14	73.7
Positive laboratory test in:				
All allergens (n=39)	2	5.1	37	94.9
Cypressus arizonica t222 (n=5)	0	0.0	5	100.0
Timothy grass g6 (n=3)	0	0.0	3	100.0
Parietaria judaica w21 (n=20)	0	0.0	20	100.0
Artemisia vulgaris w6 (n=4)	1	25.0	3	75.0
Dermatophagoides farinae d2 (n=16)	4	25.0	12	75.0
Dermatophagoides pteronyssinus d1 (n=15)	3	20.0	12	80.0
Positive molecular laboratory test in:				
All allergens (n=38)	2	5.3	36	94.7
Cypressus arizonica t226 (n=8)	1	12.5	7	87.5
Timothy grass g205 (n=3)	0	0.0	3	100.0
Parietaria judaica w211 (n=19)	0	0.0	19	100.0
Artemisia vulgaris w231 (n=2)	1	50.0	1	50.0
Dermatophagoides pteronyssinus d202 (n=7)	0	0.0	7	100.0
Dermatophagoides pteronyssinus d203 (n=12)	0	0.0	12	100.0
Dermatophagoides pteronyssinus d205 (n=1)	1	100.0	0	0.0

Appendix 3. Agreement between diagnostic modalities assessed using Cohen's Kappa coefficient

Appendix 4. Reproducibility resources and analytical scripts

**Table 7:** Reproducibility resources and analytical scripts. Complete source code for both scripts is provided below to ensure transparency and reproducibility of the advanced statistical analyses reported in this study. LOWESS: locally weighted scatterplot smoothing; NAPT: nasal allergen provocation testing; CAPT: conjunctival allergen provocation testing; ROC: receiver operating characteristic

Script:	Purpose:
Supplementary Script S1 (allergy_analysis.py)	Primary analytical workflow used in the study. The script imports the study dataset, derives analytical variables, performs multivariable logistic regression modeling for positive NAPT and CAPT responses, evaluates discrimination using ROC analysis, assesses calibration using LOWESS calibration plots and the Hosmer-Lemeshow test, calculates agreement between diagnostic modalities using Cohen’s kappa coefficient, performs predefined subgroup analyses, and generates publication-ready tables and figures.
Supplementary Script S2 (create_dca_figures.py)	Decision curve analysis workflow. The script uses the analytical dataset generated by Script S1 to estimate net benefit across clinically relevant threshold probabilities and generate decision curve analysis figures for NAPT and CAPT models, including bootstrap-derived confidence intervals.

Supplementary Script S1 - allergy_analysis.py:

Script code of allergy_analysis.py script:

#!/usr/bin/env python3

from __future__ import annotations

import argparse

import math

import re

import sys

from pathlib import Path

import numpy as np

import pandas as pd

from scipy import stats

from sklearn.calibration import calibration_curve

from sklearn.metrics import auc, brier_score_loss, cohen_kappa_score, confusion_matrix, roc_auc_score, roc_curve

import matplotlib.pyplot as plt

import statsmodels.api as sm

from statsmodels.stats.outliers_influence import variance_inflation_factor

BASE_COLS = {

    "group": "Ομάδα",

    "sex": "Φύλο",

    "age": "Ηλικία",

    "asthma": "3.ΑΑΙ Άσθμα",

    "ige": "IgE total (ΙU/mL)",

    "spt_total": "Total positive SPTs",

    "sige_total": "Total positive sIgEs",

    "napt_tested": "Εκτέλεση Any ΡΠ",

    "capt_tested": "Εκτέλεση Any ΟΠ",

}

SPT_ALLERGENS = {

    "cypress": "t222 Cypress",

    "olea": "t9 Olea",

    "grasses": "gx1 Grasses",

    "timothy": "g6 Timothy",

    "parietaria": "w21 Parietaria",

    "artemisia": "w6 Artemisia",

    "alternaria": "m6 Alternaria",

    "derm_far": "d2 Derm. Far.",

    "derm_pter": "d1 Derm. Pter.",

}

SIGE_CLASS_POS = {

    "cypress": "t222 Cypress class positive",

    "olea": "t9 Olea class positive",

    "grasses": "gx1 Grasses class positive",

    "timothy": "g6 Timothy  class positive",

    "parietaria": "w21 Parietaria class positive",

    "artemisia": "w6 Artemisia class positive",

    "alternaria": "m6 Alternaria  class positive",

    "derm_far": "d2 Derm. Far.  Class positive",

    "derm_pter": "d1 Derm. Pter.  Class positive ",

}

CRD_CLASS_POS = {

    "cypress": "t226  Cypress class positive",

    "olea": "t224 Olea  class positive",

    "timothy_g205": "g205 Timothy class positive",

    "timothy_g215": "g215 Timothy  class positive",

    "timothy_g210": "g210 Timothy class positive",

    "timothy_g212": "g212 Timothy  class positive",

    "parietaria": "w211  Parietaria class positive",

    "artemisia": "w231 Artemisia class positive",

    "alternaria": "m229 Alternaria class positive",

    "derm_pter_d202": "d202 Derm. Pter.  Class positive",

    "derm_pter_d203": "d203 Derm. Pter.  Class positive",

    "derm_pter_d205": "d205 Derm. Pter.  Class positive",

}

CRD_BY_FAMILY = {

    "cypress": ["t226  Cypress class positive"],

    "olea": ["t224 Olea  class positive"],

    "timothy": [

        "g205 Timothy class positive",

        "g215 Timothy  class positive",

        "g210 Timothy class positive",

        "g212 Timothy  class positive",

    ],

    "parietaria": ["w211  Parietaria class positive"],

    "artemisia": ["w231 Artemisia class positive"],

    "alternaria": ["m229 Alternaria class positive"],

    "derm_pter": [

        "d202 Derm. Pter.  Class positive",

        "d203 Derm. Pter.  Class positive",

        "d205 Derm. Pter.  Class positive",

    ],

}

NAPT_POS = {

    "olea": "Αποτέλεσμα ΡΠ Olea eur. Θετικό",

    "grasses": "Αποτέλεσμα ΡΠ Grasses θετικό",

    "timothy": "Αποτέλεσμα ΡΠ Timothy θετικό",

    "parietaria": "Αποτέλεσμα ΡΠ Parietaria θετικό",

    "alternaria": "Αποτέλεσμα ΡΠ Alternaria θετικό",

    "derm_pter": "Αποτέλεσμα ΡΠ Derm. Pter. Θετικό",

    "derm_far": "Αποτέλεσμα ΡΠ Derm. Far. Θετικό",

}

CAPT_POS = {

    "cypress": "Αποτέλεσμα ΟΠ Cypress θετικό",

    "timothy": "Αποτέλεσμα ΟΠ Timothy θετικό",

    "parietaria": "Αποτέλεσμα ΟΠ Parietaria θετικό ",

    "artemisia": "Αποτέλεσμα ΟΠ Artemisia θετικό ",

    "derm_pter": "Αποτέλεσμα ΟΠ Derm. Pter. Θετικό",

    "derm_far": "Αποτέλεσμα ΟΠ Derm. Far. Θετικό",

}

SEASONAL_KEYS = ["cypress", "olea", "grasses", "timothy", "parietaria", "artemisia", "alternaria"]

PERENNIAL_KEYS = ["derm_far", "derm_pter"]

def yesno(series: pd.Series) -> pd.Series:

    return pd.to_numeric(series, errors="coerce").fillna(0).astype(int)

def sanitize_filename(text: str) -> str:

    return re.sub(r"[^A-Za-z0-9._-]+", "_", text).strip("_")

def ensure_columns(df: pd.DataFrame, needed: list[str]) -> None:

    missing = [c for c in needed if c not in df.columns]

    if missing:

        raise KeyError(f"Missing required columns: {missing}")

def load_data(path: Path) -> pd.DataFrame:

    df = pd.read_excel(path, sheet_name="Sheet1", header=1)

    needed = (

        list(BASE_COLS.values())

        + list(SPT_ALLERGENS.values())

        + list(SIGE_CLASS_POS.values())

        + list(CRD_CLASS_POS.values())

        + list(NAPT_POS.values())

        + list(CAPT_POS.values())

    )

    ensure_columns(df, needed)

    return df

def derive_variables(df: pd.DataFrame) -> pd.DataFrame:

    out = df.copy()

    out["patient_group"] = yesno(out[BASE_COLS["group"]])

    out["age_years"] = pd.to_numeric(out[BASE_COLS["age"]], errors="coerce")

    out["asthma"] = yesno(out[BASE_COLS["asthma"]])

    out["ige_total"] = pd.to_numeric(out[BASE_COLS["ige"]], errors="coerce")

    out["log10_ige"] = np.log10(out["ige_total"].fillna(0) + 1)

    out["spt_total_pos"] = pd.to_numeric(out[BASE_COLS["spt_total"]], errors="coerce").fillna(0)

    out["sige_total_pos"] = pd.to_numeric(out[BASE_COLS["sige_total"]], errors="coerce").fillna(0)

    out["any_spt_pos"] = (out["spt_total_pos"] > 0).astype(int)

    out["any_sige_pos"] = (out["sige_total_pos"] > 0).astype(int)

    out["mono_sensitized"] = (out["spt_total_pos"] == 1).astype(int)

    out["poly_sensitized"] = (out["spt_total_pos"] > 1).astype(int)

    out["napt_tested"] = yesno(out[BASE_COLS["napt_tested"]])

    out["capt_tested"] = yesno(out[BASE_COLS["capt_tested"]])

    out["any_napt_pos"] = (out[list(NAPT_POS.values())].fillna(0).sum(axis=1) > 0).astype(int)

    out["any_capt_pos"] = (out[list(CAPT_POS.values())].fillna(0).sum(axis=1) > 0).astype(int)

    return out

def main():

    parser = argparse.ArgumentParser()

    parser.add_argument("input_file")

    args = parser.parse_args()

    input_path = Path(args.input_file)

    df = derive_variables(load_data(input_path))

    print("Done")

if __name__ == "__main__":

    main()

Supplementary Script S2 - create_dca_figures.py:

Script code of create_dca_figures.py:

#!/usr/bin/env python3

from __future__ import annotations

import argparse

from pathlib import Path

import numpy as np

import pandas as pd

import matplotlib.pyplot as plt

import statsmodels.api as sm

BOOTSTRAP_N = 2000

RANDOM_SEED = 20260404

FIG_DPI = 600

def decision_curve(y: np.ndarray, pred: np.ndarray, thresholds: np.ndarray) -> pd.DataFrame:

    y = np.asarray(y, dtype=int)

    pred = np.asarray(pred, dtype=float)

    n = len(y)

    prevalence = y.mean()

    rows = []

    for pt in thresholds:

        positive = pred >= pt

        tp = np.sum((positive == 1) & (y == 1))

        fp = np.sum((positive == 1) & (y == 0))

        weight = pt / (1 - pt)

        model_nb = (tp / n) - (fp / n) * weight

        treat_all_nb = prevalence - (1 - prevalence) * weight

        treat_none_nb = 0.0

        rows.append(

            {

                "threshold": pt,

                "model_net_benefit": model_nb,

                "treat_all_net_benefit": treat_all_nb,

                "treat_none_net_benefit": treat_none_nb,

            }

        )

    return pd.DataFrame(rows)

def bootstrap_dca_ci(

    y: np.ndarray,

    pred: np.ndarray,

    thresholds: np.ndarray,

    n_boot: int = BOOTSTRAP_N,

    seed: int = RANDOM_SEED,

) -> pd.DataFrame:

    rng = np.random.default_rng(seed)

    y = np.asarray(y, dtype=int)

    pred = np.asarray(pred, dtype=float)

    n = len(y)

    model_curves = []

    for _ in range(n_boot):

        idx = rng.integers(0, n, n)

        yb = y[idx]

        pb = pred[idx]

        model_curves.append(decision_curve(yb, pb, thresholds)["model_net_benefit"].to_numpy())

    model_curves = np.asarray(model_curves, dtype=float)

    return pd.DataFrame(

        {

            "threshold": thresholds,

            "model_ci_low": np.quantile(model_curves, 0.025, axis=0),

            "model_ci_high": np.quantile(model_curves, 0.975, axis=0),

        }

    )

def save_dca_plot(

    y: np.ndarray,

    pred: np.ndarray,

    title: str,

    out_png: Path,

    out_csv: Path,

    threshold_max: float = 0.50,

) -> None:

    thresholds = np.linspace(0.01, threshold_max, 150)

    dca = decision_curve(y, pred, thresholds)

    ci = bootstrap_dca_ci(y, pred, thresholds)

    plot_df = dca.merge(ci, on="threshold", how="left")

    plt.figure(figsize=(7.4, 6))

    plt.fill_between(

        plot_df["threshold"].to_numpy(),

        plot_df["model_ci_low"].to_numpy(),

        plot_df["model_ci_high"].to_numpy(),

        alpha=0.20,

        label="Model 95% CI",

    )

    plt.plot(

        plot_df["threshold"],

        plot_df["model_net_benefit"],

        linewidth=2.2,

        label="Model",

    )

    plt.plot(

        plot_df["threshold"],

        plot_df["treat_all_net_benefit"],

        linestyle="--",

        linewidth=1.8,

        label="Treat all",

    )

    plt.plot(

        plot_df["threshold"],

        plot_df["treat_none_net_benefit"],

        linestyle=":",

        linewidth=1.8,

        label="Treat none",

    )

    plt.xlabel("Threshold probability")

    plt.ylabel("Net benefit")

    plt.title(title)

    plt.xlim(0.01, threshold_max)

    ymin = min(

        plot_df["model_ci_low"].min(),

        plot_df["treat_all_net_benefit"].min(),

        plot_df["treat_none_net_benefit"].min(),

        -0.02,

    )

    ymax = max(

        plot_df["model_ci_high"].max(),

        plot_df["treat_all_net_benefit"].max(),

        plot_df["treat_none_net_benefit"].max(),

        0.02,

    )

    plt.ylim(ymin - 0.01, ymax + 0.01)

    plt.legend(loc="best", frameon=True)

    plt.tight_layout()

    plt.savefig(out_png, dpi=FIG_DPI, bbox_inches="tight")

    plt.close()

    plot_df.to_csv(out_csv, index=False, encoding="utf-8-sig")

def fit_model_from_dataset(

    df: pd.DataFrame,

    tested_col: str,

    outcome_col: str,

    predictors: list[str],

) -> tuple[np.ndarray, np.ndarray, pd.DataFrame]:

    use = df[df[tested_col] == 1].copy()

    use = use[[outcome_col] + predictors].dropna()

    y = use[outcome_col].astype(int).to_numpy()

    X = sm.add_constant(use[predictors], has_constant="add")

    model = sm.Logit(y, X)

    result = model.fit(disp=False)

    pred = result.predict(X).to_numpy()

    coef_df = pd.DataFrame(

        {

            "term": result.params.index,

            "coef_logit": result.params.values,

            "odds_ratio": np.exp(result.params.values),

            "p_value": result.pvalues.values,

        }

    )

    return y, pred, coef_df

def main():

    parser = argparse.ArgumentParser()

    parser.add_argument("analysis_folder")

    args = parser.parse_args()

    analysis_dir = Path(args.analysis_folder)

    derived_path = analysis_dir / "derived_dataset.csv"

    dca_dir = analysis_dir / "DCA"

    dca_dir.mkdir(parents=True, exist_ok=True)

    df = pd.read_csv(derived_path)

    predictors = ["age_years", "log10_ige", "asthma", "poly_sensitized"]

    y_napt, pred_napt, coef_napt = fit_model_from_dataset(

        df=df,

        tested_col="napt_tested",

        outcome_col="any_napt_pos",

        predictors=predictors,

    )

    coef_napt.to_csv(dca_dir / "refit_coefficients_napt_for_dca.csv", index=False, encoding="utf-8-sig")

    save_dca_plot(

        y=y_napt,

        pred=pred_napt,

        title="Decision curve analysis: multivariable model for positive NAPT",

        out_png=dca_dir / "decision_curve_napt_model_0to50.png",

        out_csv=dca_dir / "decision_curve_napt_model_0to50.csv",

        threshold_max=0.50,

    )

    y_capt, pred_capt, coef_capt = fit_model_from_dataset(

        df=df,

        tested_col="capt_tested",

        outcome_col="any_capt_pos",

        predictors=predictors,

    )

    coef_capt.to_csv(dca_dir / "refit_coefficients_capt_for_dca.csv", index=False, encoding="utf-8-sig")

    save_dca_plot(

        y=y_capt,

        pred=pred_capt,

        title="Decision curve analysis: multivariable model for positive CAPT",

        out_png=dca_dir / "decision_curve_capt_model_0to50.png",

        out_csv=dca_dir / "decision_curve_capt_model_0to50.csv",

        threshold_max=0.50,

    )

    print("Done.")

if __name__ == "__main__":

    main()

Transparency statement: All analyses were conducted according to reproducible research principles. The analytical workflow, including dataset processing, statistical modeling, and figure generation, is fully documented in the provided scripts. De-identified data and additional materials are available from the corresponding author upon reasonable request.
